# Séroprévalence de *Toxoplasma gondii* chez le poulet de la région de Marrakech-Safi, Maroc

**DOI:** 10.48327/mtsi.v5i1.2025.633

**Published:** 2025-01-17

**Authors:** Laila HOUMMADI, Salma BERROUCH, Oussama DEHHANI, Denis LIMONNE, Pierre FLORI, Redouane MOUTAJ, Jamal Eddine HAFID

**Affiliations:** 1 Laboratoire bioressources et sécurité sanitaire des aliments, Faculté des sciences et techniques, Université Cadi Ayyad, Marrakech, Maroc; 2 Institut supérieur des professions infirmières et techniques de santé, Marrakech, Maroc; 3 École supérieure de technologie d'El Kelaa des Sraghna, Université Cadi Ayyad, El Kelaa des Sraghna, Maroc; 4 Laboratoire LDBIO Diagnostics, Lyon, France; 5 Laboratoire de parasitologie-mycologie du Centre hospitalier universitaire (CHU) de Saint-Etienne, France; 6 Service de parasitologie-mycologie, Hôpital militaire Avicenne, Université Cadi Ayyad, Marrakech, Maroc

**Keywords:** *Toxoplasma gondii*, Poulet, ELISA, Séroprévalence, Marrakech-Safi, Maroc, Afrique du Nord, *Toxoplasma gondii*, Chicken, ELISA, Seroprevalence, Marrakech-Safi, Morocco, Northern Africa

## Abstract

**Introduction:**

*Toxoplasma gondii (T. gondii)* est un parasite intracellulaire obligatoire qui infecte un large spectre d'espèces animales dont l'homme et les animaux d'élevage. La contamination peut avoir des conséquences sanitaires, économiques et épidémiologiques considérables. Les oiseaux en général et la volaille en particulier semblent jouer un rôle important dans l'épidémiologie et la circulation du parasite. L'objectif de ce travail est de mesurer, pour la première fois, la séroprévalence de *T. gondii* chez le poulet dans la région de Marrakech-Safi.

**Matériel et méthodes:**

Les sérums ont été prélevés, entre janvier 2019 et mars 2020, sur 486 poulets provenant de trois types d'élevage : 122 poulets d'élevage traditionnel (domestique), 109 poulets fermiers et 255 poulets d'élevage commercial (en batterie) destinés à la consommation dans la région de Marrakech-Safi. La recherche des immunoglobulines Y (IgY) a été réalisée par ELISA en utilisant un antigène total de *T. gondii.*

**Résultats:**

La séroprévalence moyenne de *T.gondii* chez les poulets de la région d'étude est de 30,65 %. Cette étude a aussi montré une association significative (p<0,0001) entre la séroprévalence et le type d'élevage : le poulet domestique a une séroprévalence plus élevée que le poulet fermier et le poulet commercial.

La séropositivité élevée chez le poulet s'expliquerait par une large présence des oocystes et/ou de kystes de *T. gondii* dans son environnement et dans son alimentation.

**Conclusion:**

La consommation des produits avicoles, peu ou pas cuits, peut être une source de contamination potentielle pour l'homme, ainsi que pour les prédateurs dont le chat.

## Abstract (en)

**Introduction:**

*Toxoplasma gondii (T. gondii)* is an obligate intracellular parasite that infects a wide range of animal species, including humans and livestock. Contamination can have significant health, economic and epidemiological consequences. Birds in general, and poultry in particular, appear to play an important role in the epidemiology and circulation of the parasite. The aim of this study was to determine, for the first time, the seroprevalence of *T.gondii* in chicken in the Marrakech-Safi region.

**Materials and methods:**

Sera were collected between January 2019 and March 2020 from 486 chickens from three types of farming: 122 traditional (domestic) chickens, 109 free-range chickens, and 255 commercial (battery) chickens intended for consumption in the Marrakech-Safi region. Immunoglobulin Y (IgY) testing was performed by ELISA using a total *T. gondii* antigen.

**Results:**

The mean seroprevalence of *T.gondii* in chicken in the study region was 30.65%. This study also showed a significant association (p<0.0001) between seroprevalence and type of farming: domestic chickens had a higher seroprevalence than free-range and commercial chickens.

The high seropositivity in chicken could be explained by the widespread presence of *T. gondii* oocysts and/or cysts in their environment and diet.

**Conclusion:**

Consumption of undercooked or uncooked poultry products may be a source of potential contamination for humans and carnivores, including cats.

## Introduction

*Toxoplasma gondii* est un parasite intracellulaire obligatoire qui infecte un large spectre d'espèces animales dont l'humain et les animaux d'élevage [4, 18] ainsi que certains mammifères marins, poissons et reptiles [4, 5, 27]. Les hôtes définitifs sont les félidés domestiques et sauvages. Il cause la toxoplasmose, parasitose souvent asymptomatique et bénigne, mais qui peut être sévère chez l'individu immunodéprimé, le fœtus et le nouveau-né après transmission congénitale. En dehors de toute immunodépression, des formes graves peuvent être exceptionnellement observées avec des souches de virulence et de génotype particuliers [4, 18, 42, 52].

Les êtres humains et les animaux s'infectent le plus souvent par voie orale (à la suite de l'ingestion de viande contenant des kystes), ou par le sol, l'eau et les végétaux souillés par les fèces de chats ou d'autres félidés contenant des oocystes [4, 17, 38, 51]. En élevage, la toxoplasmose entraîne des pertes économiques, comme c'est le cas chez les ovins (perte reproductive). Elle facilite également la transmission zoonotique par la consommation de viande infectée [22, 51, 54]. Sa prévalence est variable selon les espèces. Elle est plus élevée chez les ovins et les caprins que chez les autres animaux (bovins, volailles, chiens et chevaux) [1, 20, 52]. Chez le mouton, contrairement au bovin, elle se traduit par une grande fréquence d'avortements [44, 54]. Chez ces 2 espèces, la séroprévalence varie entre 1,4 et 71 % en fonction du contexte épidémiologique et des techniques de dépistage utilisées [3, 11, 12, 13, 31, 32, 34, 37, 45, 47, 49, 53, 56].

La séroprévalence de *T.gondii* chez le poulet a fait l'objet de peu d'études dans le monde, bien qu'il soit un hôte important dans l'épidémiologie de l'infection en se contaminant généralement avec les oocystes excrétés par les chats et très résistants dans l'environnement [1]. En se nourrissant au sol, la volaille est fortement exposée à la contamination par les oocystes. C'est pour cette raison qu'elle peut être considérée comme un bon indicateur de la contamination tellurique par les oocystes [18, 23]. D'après Dubey *et al.*, la présence de kystes de *T. gondii* dans la cervelle, le cœur et les muscles du poulet peut constituer une source de contamination pour les chats [21]. Les volailles domestiques sont rarement sujettes à des formes sévères de toxoplasmose à la suite de leur infection [16, 62]. Elles développent généralement une infection chronique aux différentes souches de *T. gondii*. De ce fait, les souches de *T. gondii* qui les infectent reflètent vraisemblablement les souches qui circulent à l'échelle locale [16, 19, 36].

La séroprévalence chez le poulet varie d'un pays à un autre. Plusieurs enquêtes récentes dans des élevages domestiques ont rapporté des séropré-valences variables, parfois très élevées comprises entre 6 et 98 % [10, 29, 39, 48], ce qui montre une hétérogénéité de la séropositivité globale en fonction des pays et des méthodes de dépistage.

Au Maroc, aucune étude séro-épidémiologique n'a été réalisée à ce jour sur la prévalence de *T. gondii* chez le poulet. C'est la raison pour laquelle nous avons cherché à la déterminer dans trois types d'élevages de poulets destinés à la consommation dans la région de Marrakech-Safi (le poulet d'élevage domestique, le poulet fermier et le poulet d'élevage intensif en batterie) pour estimer le risque de contamination par l'ingestion des kystes présents dans leur chair mal cuite.

## Matériels et méthodes

L'antigène a été préparé selon le protocole de Hafid *et al.* [30], et fourni gracieusement par le laboratoire de Parasitologie-Mycologie de Saint-Etienne (France) et le laboratoire pharmaceutique LDBIO Diagnostics. Brièvement, les trophozoïtes de la souche RH de *T. gondii* ont été obtenus à partir d'ascites de souris infectées quelques jours auparavant et lavés trois fois par centrifugation à 200 g pendant 10 min dans une solution saline isotonique. Le dernier culot a été mis en suspension dans de l'eau distillée et la concentration en protéines a été dosée et fixée par dilution à 1 mg/ml. La solution finale a été considérée comme un antigène total.

**Figure 1:**
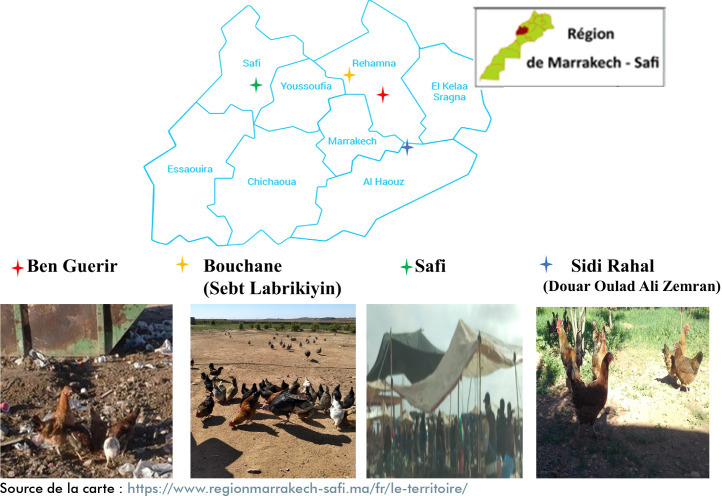
Localisation géographique des zones d'échantillonnage des poulets domestiques. ©Hoummadi Laila.

Dans cette étude, nous avons prélevé 486 sérums provenant de trois types de poulets :

- Cent vingt-deux poulets d'environ un an provenant d'élevages domestiques de plein air, sans clôture, se nourrissant en picorant le sol et consommant divers aliments, y compris des céréales et des déchets ménagers. Des échantillons de sang ont été prélevés sur ces poulets, qui ont ensuite été relâchés sans être abattus. Sept campagnes de prélèvements ont été réalisées entre janvier 2019 et mars 2020, sur quatre sites respectivement à Ben Guerir (n=17), Bouchane (n=36), Sidi Rahal (n=35), et Safi (n=34) (Fig. 1). L'échantillonnage de ce type de poulets a été réalisé avec l'accord de leurs propriétaires.
- Cent neuf poulets fermiers provenant des élevages contrôlés et vendus dans les abattoirs de la ville de Marrakech. Ces poulets ont été élevés en plein air dans des fermes pendant une durée maximum de 71 jours durant laquelle ils ont bénéficié d'une alimentation à base de céréales et de végétaux. Ces poulets disposaient d'un espace clôturé suffisamment grand pour qu'il y ait une densité de 11 poulets/ m^2^ (Arrêté du ministère de l'agriculture marocain n°1271-17), et protégé de l'accès des chats. Le sang de ces poulets a été collecté entre janvier 2019 et mars 2020, au niveau de deux abattoirs choisis arbitrairement dans la ville de Marrakech.
- Deux cent cinquante-cinq poulets d'élevage commercial, confinés et âgés de 35 à 40 jours. Ces animaux provenaient des élevages en batterie et se nourrissaient d'une alimentation à base de céréales, de tourteaux, d'oléagineux et de farine de poisson. Ces volailles ont été élevées dans des bâtiments clôturés, mises sur litière en paille, et ont disposé d'un espace suffisant pour circuler en liberté, sans accès à l'extérieur.

Le sang de ces poulets a été prélevé, entre janvier 2019 et mars 2020, dans quatre abattoirs choisis arbitrairement dans la ville de Marrakech. Le sang a été collecté dans des tubes sans anticoagulant à partir de la veine alaire pour le poulet d'élevage traditionnel et directement de la veine jugulaire au moment de l'abattage pour les deux autres types de poulet dans les abattoirs de la ville de Marrakech. Les échantillons ont été centrifugés à 200 g pendant 30 min et les sérums testés par ELISA pour la recherche des IgY anti-T *gondii.* Quatre poulets d'élevage commercial âgés de 40 jours et pesant entre 1,5 et 2 kg ont été achetés dans le commerce et utilisés pour produire des sérums hyper-immuns anti-T. *gondii.* Avant leur immunisation, et pour vérifier leur statut immunitaire vis-à-vis de *T. gondii*, des prélèvements sanguins ont été réalisés au niveau de leurs veines alaires. Les résultats des tests en ELISA et en immunoblot ont montré que les quatre poulets étaient négatifs.

L'immunisation a été réalisée selon le protocole préconisé par Vaitukaitis *et al.* [57]. Un mélange contenant 1 ml d'antigène total (AT) de *T. gondii* et 1 ml d'adjuvant complet de Freund (Thermo scientific) a été injecté par voie intradermique, au niveau du flanc de chaque poulet. Dix jours après, une injection de rappel a été faite dans les mêmes conditions. Les animaux ont été gardés dans un poulailler et la production des IgY anti-T. *gondii* a été recherchée à J0, J10, J31, J41, J60 par ELISA et immunoblot.

Les sérums de 10 poulets d'élevage commercial, sélectionnés parmi les 486 poulets étudiés, ayant montré une très faible densité optique (DO) lors des essais de l'ELISA et donné des résultats négatifs en immunoblot, ont été considérés comme témoins négatifs.

Pour déterminer le seuil de positivité, nous avons ajouté trois déviations standard à la moyenne arithmétique des DO de ces sérums. Le seuil retenu est 0,260.

Les puits en polystyrène de la plaque ELISA (Nunc, Merck) ont été sensibilisés en ajoutant 100 µl d'AT à une concentration de 20 µg de protéine par millilitre. La plaque a été incubée pendant 1 heure à 37 °C, puis toute la nuit à 4 °C. Ensuite, les puits ont été lavés trois fois pendant 5 minutes avec une solution de tampon phosphate salin (PBS) contenant du Tween 20 à 0,05 %. Après une saturation d'une heure avec du PBS-Tween 20 et 5 % d'albumine sérique bovine (BSA), les sérums dilués dans la même solution ont été déposés à raison de 100 µl par puits et incubés pendant 2 heures à 37 °C. Après avoir lavé les puits comme décrit ci-dessus, 100 µl d'anticorps IgY de poulet couplé à la peroxydase (Sigma), préalablement dilués à 1/500 dans du PBS-Tween 20 et 0,5 % de BSA, ont été ajoutés dans les puits et incubés pendant 1 heure à 37 °C. Après le lavage des puits, la réaction a été révélée en ajoutant 100 µl de substrat. Ce dernier a été obtenu en dissolvant un comprimé de dihydrochlorure d'O-phénylè-nediamine (Sigma) dans 20 ml de tampon citrate complété avec 26 µl de H_2_O_2_ à 30 %. Les plaques ont été maintenues dans l'obscurité pendant 15 minutes, puis la densité optique a été lue avec un spectrophotomètre à 492 nm (BioTeK ELx800). La reproductibilité a été vérifiée en testant dans chaque nouvelle série huit sérums positifs et huit sérums négatifs de la série précédente.

La détection des IgY anti-*T. gondii* par immunoblot chez les poulets immunisés avec l'AT a été réalisée par le laboratoire LDBIO-Diagnostics. Brièvement, chaque échantillon à tester a été incubé séparément avec une bandelette contenant l'AT. Après lavages, les anti-IgY marqués à la phosphatase alcaline (LDBIO) ont été déposés sur chaque bandelette. L'incubation et les lavages ont été suivis par l'ajout du substrat qui entraîne, lorsque l'échantillon est positif, l'apparition de bandes de poids moléculaire 30, 31, 33, 40 et 45 kDa. La présence d'au moins 3 bandes parmi les 5, incluant la bande à 30 kDa, permet d'affirmer la présence d'anticorps IgY anti-T. *gondii* dans le sérum analysé.

Tous les animaux ont été traités selon les règles d'éthique en vigueur et tous les prélèvements ont été réalisés avec l'aval des autorités sanitaires locales et des propriétaires de poulets auxquels nous avons expliqué amplement les raisons et les objectifs de nos travaux.

L'analyse descriptive a consisté au calcul des fréquences absolues des variables qualitatives, aux paramètres de positionnement et à la dispersion des variables quantitatives (moyenne, écart-type). La distribution normale des variables a été étudiée par le test de Kolmogorov-Smirnov. En analyse bivariée, la comparaison des variables qualitatives a fait appel au test statistique de Chi^2^ de Pearson et à celui de Fisher si nécessaire. Le seuil de significativité était retenu pour p<0,05. L'analyse statistique a été effectuée à l'aide du logiciel SPSS version 19.0.

## Résultats

Les IgY anti-*T. gondii* ont été détectées à partir du dixième jour (J10) de l'immunisation aussi bien en ELISA qu'en immunoblot. Ces anticorps persistent et leur titre augmente jusqu'à J70, jour de la récupération de tout le sang corporel des animaux. Ces sérums sont considérés comme témoins positifs pour l'étude de la prévalence des IgY anti-T. *gondii*, par ELISA, chez les différents types de poulets de notre étude.

En adoptant l'ELISA, nous avons obtenu une prévalence globale de *T. gondii* de 30,65 % chez le poulet de la région de Marrakech-Safi. Elle est de 40,2 % chez le poulet d'élevage traditionnel, de 10,1 % chez le poulet fermier et de 34,9 % chez le poulet d'élevage commercial en batterie (Tableau I). Cette différence de séroprévalence par rapport aux types d'élevage est significative (p<0,0001).

**Tableau I:** Séroprévalence de *T. gondii* chez les poulets de la région de Marrakech-Safi

Animal	Nombre d'échantillons testés	Nombre de positifs	Prévalence	p
**Poulet d'élevage traditionnel (domestique)**	122	49	40,2 %	0,0001
**Poulet fermier**	109	11	10,1 %	
**Poulet d'élevage commercial**	255	89	34,9 %	
**Prévalence moyenne**			30,7 %	

Pour le poulet domestique, la séroprévalence est variable selon la ville de provenance : 76,5 % à Ben Guerir, 52,9 % à Safi, 31,4 % à Sidi Rahal et 19,4 % à Bouchane (Tableau II).

**Tableau II:** Séroprévalence de *T. gondii* chez les poulets domestiques selon les villes de la région Marrakech-Safi

Ville	Nombre d'échantillons testés	Nombre de positifs	Prévalence	p
**Ben Guerir**	17	12	76,5 %	0,0001
**Safi**	34	18	52,9 %	
**Bouchane**	36	7	19,4 %	
**Sidi Rahal**	35	11	31,4 %	

## Discussion

Au Maroc, à l'instar de plusieurs pays, le poulet joue un rôle considérable dans la vie quotidienne, culturelle et sociale des citoyens. Selon le ministère de l'agriculture, il est la première source de protéine animale, et est utilisé dans la majorité des cérémonies rituelles et religieuses. Ces animaux sont généralement élevés et abattus dans des conditions non surveillées, à domicile ou chez des bouchers traditionnels, pour la consommation locale. Les viscères, les têtes ainsi que les restes de viande sont jetés dans les poubelles publiques et sont facilement récupérés et consommés par les animaux errants (chats, chiens, rats…). Ceci peut représenter une source non négligeable de circulation des souches de *T. gondii* entre les différents hôtes.

Malgré une aire de circulation réduite par rapport à d'autres animaux, le poulet domestique partage souvent son environnement avec les chats. Son comportement alimentaire, caractérisé par un picorage au sol pour la recherche de nourriture, augmente son exposition aux déjections des chats potentiellement contaminées par des oocystes et enfouies dans le sol [21]. Ainsi, Dubey *et al.* [19] et Lehmann *et al.* [36] ont considéré le poulet comme un bon indicateur de la contamination tellurique par les oocystes.

Certaines études ont montré que les kystes de *T. gondii* se localisent principalement dans le cœur et à un degré moindre dans le cerveau et les autres viscères, mais plus rarement dans les muscles striés [2, 22, 24, 31, 35]. La consommation d'un poulet insuffisamment cuit représente une importante source d'infection pour les différents hôtes dont l'humain et le chat [1, 14].

Ce travail, le premier au Maroc, et dans la région de Marrakech-Safi en particulier, montre que la séroprévalence moyenne anti-*T. gondii* est de 30,7 %, ce qui indique que l'infection chez les poulets est largement répandue dans la région et qu'elle varie grandement selon le type d'élevage. Elle est plus élevée dans l'élevage traditionnel (40,2 %) que dans l'élevage commercial (34,9 %) et l'élevage fermier (10,1 %), ce qui confirme le constat rapporté par plusieurs études [10, 26, 40, 41, 46, 56, 59]. Ceci suggère que les poulets domestiques élevés en liberté, en contact étroit avec les chats, ont un risque beaucoup plus élevé de contracter une infection à *T. gondii* que les poulets de chair confinés. Par ailleurs, la durée de vie des poulets – 1 an pour les poulets domestiques, 71 jours pour les poulets fermiers et 35-40 jours pour les poulets d'élevage intensif – pourrait contribuer à l'accumulation du parasite et expliquer une prévalence plus élevée. Toutefois, la prévalence chez le poulet d'élevage intensif, qui est de 34,9 %, reste élevée par rapport à celles retrouvées chez le même type de poulet au centre du Portugal 5,6 % (10/178) par test d'agglutination modifié (MAT, *modified agglutination test)* [46] et nulle dans la ville de Gifu en Chine (0/163) par test d'agglutination au latex (LAT, *latex agglutination test)* [41]. C'est un constat inhabituel car, dans ce mode d'élevage, les prévalences de l'infection par *T. gondii* sont souvent très faibles, voire nulles, du fait que le poulet est abrité dans des bâtiments protégés de l'accès des chats, et qu'il se nourrit d'une alimentation à base de farine.

Une visite dans les fermes des poulets d'élevage intensif de la région nous a permis de constater que les normes dans ce type d'exploitation ne sont pas respectées. Plusieurs indices indiquant clairement que les fermes manquent au respect des règles d'hygiène ont été décelés. Il est toutefois clair que les principaux facteurs à considérer en lien avec la séroprévalence élevée chez ce type de poulet sont davantage liés à :

- la maîtrise insuffisante du confinement en raison de l'état de la clôture grillagée qui ne permet pas d'empêcher la pénétration des chats;
- l'ouverture des locaux de stockage sur l'extérieur. Cette ouverture favorise l'accès aux oiseaux, rongeurs et insectes nuisibles, agents de dispersion mécanique de parasite [28];
- l'utilisation de la paille qui peut être potentiellement souillée par des fèces de chats contenant des oocystes et/ou carcasses de rongeurs renfermant des kystes de *T. gondii* et qui maintiendrait une prévalence élevée de l'infection [14];
- la non disponibilité d'un programme de lutte contre les nuisibles et la mauvaise gestion des carcasses;
- une contamination possible du sol avant l'installation de la ferme ou encore la contamination du milieu d'élevage pendant la période du vide sanitaire;
- l'utilisation de l'eau de puits qui peut être contaminée par les parasites dont *T. gondii.*

En plus de ces facteurs, il ne faut pas écarter l'éventualité de la présence de chats errants qui souillent souvent les entrepôts de nourriture par leurs fèces contenant les oocystes de *T. gondii.* Il suffit probablement d'une seule fèce pour contaminer tout le terrain [7]. La consommation de viande de poulets issus d'élevage intensif est plus élevée que celle des autres types de poulet, augmentant ainsi l'importance de ce type de volaille comme source potentielle d'infection. La compréhension actuelle de l'épidémiologie de la toxoplasmose nous amène à penser que ce type de poulet contracte l'infection suite à la consommation d'aliments et d'eau contaminés par les oocystes de *T. gondii* excrétés par les chats. Concernant le poulet fermier, la prévalence de l'infection à *T. gondii* dans l'élevage est de 10,1 %. Elle est faible par rapport à celle des autres types d'élevage, ce qui est probablement dû aux strictes pratiques de protection et d'hygiène exigées par l'arrêté du ministère de l'agriculture marocain (n°1271-17) portant reconnaissance du label agricole (poulet fermier) et l'homologation du cahier des charges y afférant. Ces pratiques comprennent des contrôles réguliers des conditions d'élevage, des mesures pour éviter la contamination des aliments et de l'eau, ainsi que des protocoles de nettoyage et de désinfection. Ces mesures contribuent à réduire la contamination par *T.gondii.* Bien que les poulets fermiers aient accès à l'extérieur, ils sont souvent élevés dans des environnements où les sources de contamination sont mieux contrôlées que dans les élevages intensifs.

Le risque de transmission de *T. gondii* par la consommation de viande issue de ce type d'élevage est faible, mais il n'est pas nul. La contamination des poulets par le parasite reste possible, même avec une gestion rigoureuse de l'élevage et des pratiques d'hygiène strictes. Une source potentielle de contamination pourrait être le lessivage des sols, où les oocystes présents dans les déjections des chats pourraient être disséminés par les eaux de pluie, introduisant ainsi une faible possibilité de contamination malgré les mesures de contrôle.

Nous avons relevé également des prévalences variables, avec une différence significative (p<0.0001), selon les villes dans lesquelles nous avions pu faire des prélèvements chez les poulets élevés traditionnellement. C'est une prévalence élevée qui peut signifier que l'environnement de ces villes contient non seulement des oocystes présents dans le sol, dans l'eau et sur les végétaux [8], mais qu'il existe aussi des rongeurs, des vers de terre et des animaux porteurs des kystes et/ ou oocystes du parasite [9, 25, 58]. Puisque les poulets élevés en liberté tirent leur nourriture du sol, ils sont probablement infectés par l'ingestion d'oocystes plutôt que par de la viande contenant des kystes [33, 61].

En effet, dans les villes de Ben Guerir et de Safi où la séroprévalence est de respectivement 76,5 % et 52,9 %, nous avions noté, durant nos sept campagnes de prélèvements, que les poulets se déplaçaient librement pour trouver leur propre nourriture. Ils se nourrissaient souvent dans des poubelles publiques et des endroits où la présence des chats errants était très fréquente. Alors que dans les villes de Bouchane (19,4 %) et de Sidi Rahal (31,4 %) les poulets se nourrissaient de céréales, de pain et de déchets de légumes fournis par l'éleveur. Le contact avec les chats errants était moins fréquent que dans les deux villes précédentes.

La prévalence de la toxoplasmose est très variable selon les espèces. Elle est généralement plus élevée chez le mouton, la chèvre et le porc que chez les autres animaux domestiques : bovins, volailles, chiens et chevaux [1, 20, 52]. Cependant, la séroprévalence obtenue dans notre étude montre qu'au Maroc, ce n'est pas toujours le cas. Les poulets de la région Marrakech-Safi s'avèrent plus contaminés que les ovins de la même région, qui avaient une séroprévalence de 27,6 % par ELISA en 2008 (72/261) [50]. De même en Égypte, la séroprévalence de *T. gondii* chez le poulet dans la ville de Gharbia était de 82,3 % par ELISA [6]. Elle était plus élevée que celle rapportée chez les ovins (51,8 %) (88/170) dans la même ville de Gharbia [34].

Ces résultats confirment que, pour l'agent de la toxoplasmose, la variation de la prévalence est liée non seulement aux espèces animales mais aussi, et très souvent, aux techniques d'élevage, à la qualité des ressources en eau, aux conditions d'hygiène des fermes, aux conditions climatiques et à la cohabitation avec les chats errants.

## Conclusion

La présente étude a montré une séroprévalence moyenne de 30,7 %. La positivité trouvée dans tous les élevages permet de conclure que la toxoplasmose est répandue chez la majorité des élevages de poulets, même chez les poulets confinés. Une association significative a pu être mise en évidence entre la séroprévalence de *T. gondii* et le type d'élevage. Les variations dans les taux d'infection semblent liées aux différences dans les méthodes de gestion. Les poulets en élevage intensif sont aussi susceptibles d'être exposés aux oocystes de *T. gondii* présents dans les pâturages et l'eau que les poulets fermiers, qui bénéficient d'une gestion plus contrôlée avec une alimentation et une eau de qualité. Une séroprévalence plus élevée a été observée chez les poulets domestiques d'un an, élevés en plein air, ce qui suggère qu'ils présentent un risque plus élevé pour le consommateur. La contamination de poulet est un reflet indirect de la contamination environnementale. C'est pour cette raison que l'on peut confirmer que la prévalence de *T. gondii* chez le poulet est un excellent indicateur pour estimer la contamination des lieux de son élevage et pouvoir endiguer la circulation des souches de toxoplasme. Les produits avicoles peuvent être une source de contamination alimentaire. En outre, le respect des règles d'hygiène durant toutes les étapes de production de la viande de volailles (élevage, abattage et commercialisation) est primordial pour préserver les qualités sanitaires du produit et protéger la santé des consommateurs de viande peu ou pas cuite.

## Source de financement

Ce travail n'a bénéficié d'aucune source de financement.

## Contribution des auteurs et autrices

Laila Hoummadi : Prélèvement, méthodologie, rédaction de l'article et validation.

Salma Berrouch et Oussama Dehhani : Prélèvement et validation.

Denis Limonne et Pierre Flori : conceptualisation, validation et ressources.

Redouane Moutaj et Jamal Eddine Hafid : supervision et correction de l'article.

## Liens d'intérêts

Les auteurs déclarent ne pas avoir de liens d'intérêts.
