# Supplementary material for: Seroprevalence of Toxoplasma gondii in chicken of the Marrakech-Safi region, Morocco
Source: Med Trop Sante Int. 2025 Jan 17;5(1):mtsi.v5i1.2025.633. [Article in French] doi: 10.48327/mtsi.v5i1.2025.633 (PMC12001991; doi:10.48327/mtsi.v5i1.2025.633)
Supplement: Related texts

# Research article: Seroprevalence of *Toxoplasma gondii* in chicken of the Marrakech-Safi region, Morocco

## Introduction

*Toxoplasma gondii* is an obligate intracellular parasite that infects a wide range of animal species, including humans and livestock [4, 18], as well as certain marine mammals, fish and reptiles [4, 5, 27]. The definitive hosts are domestic and wild felids. It causes toxoplasmosis, a parasitosis that is often asymptomatic and benign, but can be severe in immunocompromised individuals, fetuses and neonates after congenital transmission. In the absence of immunosuppression, severe forms may be seen in exceptional cases with strains of particular virulence and genotype [4, 18, 42, 52].

Humans and animals are most commonly infected orally (after ingestion of meat containing cysts) or through soil, water, and plants contaminated with oocyst-containing feces of cats or other felids [4, 17, 38, 51]. In livestock, toxoplasmosis causes economic losses, as in the case of sheep (loss in reproduction). It also facilitates zoonotic transmission through the consumption of infected meat [22, 51, 54]. Its prevalence varies between species. It is higher in sheep and goats than in other animals (cattle, poultry, dogs and horses) [1, 20, 52]. In sheep, in contrast to cattle, it causes a high incidence of abortion [44, 54]. In these two species, the seroprevalence varies from 1.4% to 71%, depending on the epidemiologic context and the screening techniques used [3, 11, 12, 13, 31, 32, 34, 37, 45, 47, 49, 53, 56].

Seroprevalence of *T. gondii* in chicken has been poorly studied worldwide, despite the fact that they are an important host in the epidemiology of the infection, commonly contaminated with oocysts shed by cats and highly resistant in the environment [1]. Poultry are highly exposed to oocyst contamination through ground feeding. For this reason, it can be considered as a good indicator of telluric oocyst contamination [18, 23]. According to Dubey *et al.*, the presence of *T. gondii* cysts in chicken brain, heart, and muscle may be a source of contamination for cats [21].

Poultry rarely develop severe forms of toxoplasmosis after infection [16, 62]. They typically develop chronic infection with multiple strains of *T. gondii.* As a result, the strains of *T. gondii* that infect them are likely to reflect those circulating locally [16, 19, 36].

Seroprevalence in chicken varies from country to country. Several recent surveys in domestic flocks have reported variable, sometimes very high, seroprevalences ranging from 6 to 98% [10, 29, 39, 48], showing heterogeneity in overall seropositivity depending on the country and screening method.

In Morocco, no sero-epidemiological study on the prevalence of *T. gondii* in chicken has been performed. For this reason, we set out to determine the prevalence of *T.gondii* in three types of chickens reared for consumption in the Marrakech-Safi region (domestic chickens, free-range chickens, and intensive battery chickens) in order to estimate the risk of contamination by ingestion of cysts in their undercooked meat.

## Materials and methods

The antigen was prepared according to the protocol by Hafid *et al.* [30] and kindly provided by the Parasitologie-Mycologie Laboratory of Saint-Etienne (France) and the LDBIO Diagnostics pharmaceutical laboratory. Briefly, trophozoites of the RH strain of *T. gondii* were obtained from the ascites of mice infected a few days earlier and washed three times by centrifugation at 200 g for 10 min in isotonic saline. The final pellet was suspended in distilled water and the protein concentration was determined by dilution to 1 mg/ml. The final solution was considered as total antigen.

In this study, we collected 486 sera from three types of chickens:

- One hundred and twenty-two approximately one-year-old chickens from free-range, unfenced domestic flocks that feed by pecking at the ground and consume a variety of diets, including cereals and household waste. Blood samples were collected from these chickens, which were then released without being slaughtered. Seven sampling campaigns were conducted between January 2019 and March 2020 at four sites each in Ben Guerir (n=17), Bouchane (n=36), Sidi Rahal (n=35), and Safi (n=34) (Fig. 1). These chickens were sampled with the consent of their owners.
- One hundred and nine free-range chickens from controlled farms sold in slaughterhouses in the city of Marrakech. These chickens were raised in the open air on farms for a maximum of 71 days, during which time they were fed a diet based on cereals and vegetables. The chickens were kept in a fenced area large enough to ensure a density of 11 chickens per square meter (Moroccan Ministry of Agriculture Order No. 1271-17) and protected from access by cats. The blood of these chickens was collected between January 2019 and March 2020 at two randomly selected slaughterhouses in the city of Marrakech.
- Two hundred and fifty-five commercial chickens, caged and aged between 35 and 40 days. These animals came from battery farms and were fed a diet based on cereals, oil cakes, oilseeds and fish meal. The chickens were housed in fenced buildings, on straw bedding, and given sufficient space to roam freely without access to the outdoors.

**Figure 1:** Geographic location of the sampling zones of domestic chickens. ©Hoummadi Laila

The blood of these chickens was collected between January 2019 and March 2020 at four randomly selected slaughterhouses in the city of Marrakech. Blood was collected in anticoagulant-free tubes from the wing vein for traditionally raised chickens and directly from the jugular vein at the time of slaughter for the other two types of chickens in slaughterhouses in the city of Marrakech. Samples were centrifuged at 200 g for 30 min and sera were tested by ELISA for anti-T. *gondii* IgY.

Four 40-day-old commercial chickens weighing between 1.5 and 2 kg were purchased commercially and used to prepare hyperimmune anti-T. *gondii* sera. Blood samples were taken from the wing veins prior to immunization to determine their immune status against *T. gondii.* The results of ELISA and immunoblot tests showed that all four chickens were negative.

Immunization was performed according to the protocol recommended by Vaitukaitis *et al.* [57]. A mixture containing 1 ml of *T. gondii* total antigen (TA) and 1 ml of Freund's complete adjuvant (Thermo Scientific) was injected intradermally into the flank of each chicken. Ten days later, a booster injection was given under the same conditions. Animals were housed in a poultry house and anti-T. *gondii* IgY production was assessed by ELISA and immunoblot at D0, D10, D31, D41 and D60.

Sera from 10 commercially bred chickens, selected from the 486 chickens tested, that had very low optical density (OD) in ELISA tests and negative immunoblot results were used as negative controls.

To determine the positivity threshold, three standard deviations were added to the arithmetic mean OD of these sera. The threshold used was 0.260.

The polystyrene wells of the ELISA plate (Nunc, Merck) were sensitized by adding 100 µl of AT at a concentration of 20 µg protein per milliliter. The plate was incubated at 37°C for 1 hour and then at 4°C overnight. The wells were then washed three times for 5 minutes with phosphate-buffered saline (PBS) containing 0.05% Tween 20. After saturation with PBS-Tween 20 and 5% bovine serum albumin (BSA) for 1 hour, sera diluted in the same solution were plated at 100 µl per well and incubated at 37°C for 2 hours. After washing the wells as described above, 100 µl of peroxidase-labeled chicken IgY antibody (Sigma), previously diluted 1:500 in PBS-Tween 20 and 0.5% BSA, was added and incubated for 1 hour at 37°C. After washing the wells, the reaction was visualized by adding 100 µl of substrate. The substrate was prepared by dissolving one tablet of O-phenylenediamine dihydrochloride (Sigma) in 20 ml of citrate buffer supplemented with 26 µl of 30% H_2_O_2_. Plates were kept in the dark for 15 minutes and the optical density was read using a spectrophotometer at 492 nm (BioTeK ELx800). Reproducibility was checked by testing 8 positive and 8 negative sera from the previous series in each new series.

Detection of anti-T. *gondii* IgY by immunoblot in chickens immunized with AT was carried out by LDBIO-Diagnostics. Briefly, each test sample was incubated separately with a strip containing AT. After washing, alkaline phosphatase-labeled anti-IgY (LDBIO) was applied to each strip. Incubation and washes were followed by addition of the substrate which, when the sample was positive, resulted in the appearance of bands of molecular weight 30, 31, 33, 40 and 45 kDa. The presence of at least 3 of the 5 bands, including the 30 kDa band, confirms the presence of IgY anti-*T. gondii* antibodies in the serum analyzed. All animals were treated ethically, and all samples were taken with the approval of the local health authorities and the chicken owners, to whom we gave a full explanation of the reasons for and aims of our work.

The descriptive analysis consisted in calculating the absolute frequencies of the qualitative variables, the positioning parameters and the dispersion of the quantitative variables (mean, standard deviation).

The normal distribution of the variables was studied using the Kolmogorov-Smirnov test. In bivariate analysis, qualitative variables were compared using Pearson's Chi^2^ statistical test, and Fisher's if necessary. The significance threshold was p<0.05. Statistical analysis was performed using SPSS software version 19.0.

## Results

Anti-*T*.*gondii* IgY antibodies were detected from day ten (D10) of immunization by both ELISA and immunoblot. These antibodies persisted and their titer increased until D70, the day on which all the animals’ body blood was recovered. These sera are considered as positive controls for the study of the prevalence of anti-*T. gondii* IgY, by ELISA, in the different types of chickens in our study. Using ELISA, we obtained an overall *T. gondii* prevalence of 30.65% in chicken from the Marrakech-Safi region. This compares with 40.2% in traditionally-reared (domestic) chicken, 10.1% in free-range chicken and 34.9% in commercially-reared battery chickens (Table I). The difference in seroprevalence between types of farming is significant (p<0.0001).

**Table I:** Seroprevalence of *T. gondii* in chicken in the region of Marrakesh-Safi

Animal	Number of samples tested	Number of positive samples	Prevalence	p
**Traditionally-reared (domestic) chicken**	122	49	40,2 %	0,0001
**Free-range chicken**	109	11	10,1 %	
**Commercially-reared battery chickens**	255	89	34,9 %	
**Average prevalence**			30,7 %	

For domestic chicken, seroprevalence varied according to town of origin: 76.5% in Ben Guerir, 52.9% in Safi, 31.4% in Sidi Rahal and 19.4% in Bouchane (Table II).

**Table II:** Seroprevalence of *T. gondii* in domestic farmed chicken according to the cities of the region of Marrakesh-Safi

City	Number of samples tested	Number of positive samples	Prevalence	p
**Ben Guerir**	17	12	76,5 %	0,0001
**Safi**	34	18	52,9 %	
**Bouchane**	36	7	19,4 %	
**Sidi Rahal**	35	11	31,4 %		

## Discussion

In Morocco, as in many other countries, chicken plays an important role in the daily, cultural and social life of the citizens. According to the Ministry of Agriculture, it is the main source of animal protein and is used in most ritual and religious ceremonies. These animals are generally raised and slaughtered in unsupervised conditions, at home or by traditional butchers, for local consumption. The entrails, heads and meat scraps are disposed of in public garbage cans and are easily collected and consumed by stray animals (cats, dogs, rats…). This can be a significant source of circulation of *T.gondii* strains between different hosts.

Despite their limited range compared to other animals, domestic chickens often share their environment with cats. Their feeding behavior, characterized by pecking at the ground in search of food, increases their exposure to cat feces, which may be contaminated with oocysts and buried in the soil [21]. Thus, Dubey *et al.* [19] and Lehmann *et al.* [36] considered chickens to be a good indicator of telluric contamination by oocysts.

Some studies have shown that *T. gondii* cysts are mainly found in the heart and to a lesser extent in the brain and other viscera, and less commonly in striated muscles [2, 22, 24, 31, 35]. Consumption of undercooked chicken is an important source of infection for various hosts, including humans and cats [1, 14].

This study, the first in Morocco and in particular in the Marrakech-Safi region, shows that the average anti-*T. gondii* seroprevalence is 30.7%, indicating that infection in chicken is widespread in the region and varies greatly according to the type of farming. It is higher in traditional farming (40.2%) than in commercial farming (34.9%) and free-range farming (10.1%), confirming the results of several studies [10, 26, 40, 41, 46, 56, 59]. This suggests that free-range domestic chickens in close contact with cats have a much higher risk of *T. gondii* infection than confined broilers. Furthermore, the lifespan of chickens - 1 year for domestic chickens, 71 days for free-range chickens and 35-40 days for intensive broilers - could contribute to parasite accumulation and explain the higher prevalence. However, the prevalence in intensively reared chickens, at 34.9%, remains high compared to those found in the same type of chickens in central Portugal (5.6%, 10/178) by modified agglutination test (MAT) [46] and zero in the Chinese city of Gifu (0/163) by latex agglutination test (LAT) [41]. This is an unusual finding, as the prevalence of *T. gondii* infection in this type of farming is often very low or even non-existent due to the fact that chickens are housed in buildings protected from access by cats and fed a flour-based diet.

A visit to the intensive chicken farms in the region revealed that the standards for this type of operation are not being met. There were a number of clear indications that the farms were not complying with hygiene regulations. However, it is clear that the main factors associated with the high seroprevalence in this type of chicken are more related to :

- Inadequate containment due to the condition of the wire fencing, which does not prevent cats from entering the premises;
- The openness of the warehouses to the outside. This openness facilitates access by birds, rodents and insect pests, which are agents of mechanical parasite spread [28];
- The use of straw, which may be contaminated by cat feces containing oocysts and/or rodent carcasses containing *T. gondii* cysts, thus maintaining a high prevalence of infection [14];
- Lack of a pest control program and poor carcass management;
- Possible contamination of the soil prior to the establishment of the farm, or contamination of the rearing environment during the fallow period;
- The use of well water that may be contaminated with parasites, including *T. gondii.*

In addition to these factors, the presence of stray cats, which often contaminate feed stores with feces containing *T. gondii* oocysts, cannot be excluded. A single fecal pellet is probably sufficient to contaminate the entire area [7]. The consumption of meat from intensively farmed chickens is higher than that of other kind of chicken raising, increasing the importance of this type of poultry as a potential source of infection. Current understanding of the epidemiology of toxoplasmosis suggests that these chickens become infected after consuming food and water contaminated with *T. gondii* oocysts shed by cats.

The prevalence of *T. gondii* infection in freerange chickens is 10.1%. This is low compared to other types of farming, probably due to the strict protection and hygiene practices required by the Moroccan Ministry of Agriculture's decree (n°1271-17) recognizing the agricultural label *(poulet fermier)* and approving its specifications. These practices include regular inspections of rearing conditions, measures to prevent contamination of feed and water, and cleaning and disinfection protocols. These measures help to reduce *T. gondii* contamination. Although freerange chickens have access to the outdoors, they are often raised in environments where sources of contamination are better controlled than in intensive systems.

The risk of *T. gondii* transmission through consumption of meat from this type of farming is low, but not zero. Contamination of chickens with the parasite remains a possibility, even with rigorous farm management and strict hygiene practices. A potential source of contamination could be soil leaching, where oocysts present in cat feces could be spread by rainwater, creating a small possibility of contamination despite control measures.

We also found different prevalences, with a significant difference (p<0.0001), depending on the towns where we were able to sample traditionally raised chickens. This high prevalence may mean that the environment in these towns contains not only oocysts in soil, water and plants [8], but also rodents, earthworms and animals carrying cysts and/or oocysts of the parasite [9, 25, 58]. Because free-range chickens obtain their food from the soil, they are likely to be infected by ingesting oocysts rather than meat containing cysts [33, 61]. In fact, in the towns of Ben Guerir and Safi, where the seroprevalence is 76.5% and 52.9%, respectively, we observed during our seven sampling campaigns that chickens moved freely to find their own food. They often fed in public garbage bins and in places where stray cats were common. In contrast, in the towns of Bouchane (19.4%) and Sidi Rahal (31.4%), chickens fed on grain, bread, and vegetable waste provided by the farmer. Contact with stray cats was less frequent than in the two previous towns.

The prevalence of toxoplasmosis varied considerably between species. It is generally higher in sheep, goats and pigs than in other domestic animals: cattle, poultry, dogs and horses [1, 20, 52]. However, the seroprevalence obtained in our study shows that this is not always the case in Morocco. Chickens from the Marrakech-Safi region were found to be more contaminated than sheep from the same region, which had a seroprevalence of 27.6% by ELISA in 2008 (72/261) [50]. Similarly, in Egypt, the seroprevalence of *T. gondii* in chicken in the city of Gharbia was 82.3% by ELISA [6]. It was higher than that reported in sheep (51.8%) (88/170) in the same city of Gharbia [34].

These results confirm that the variation in the prevalence of the toxoplasmosis agent is not only related to the animal species, but also, and very often, to the breeding techniques, the quality of water resources, the hygienic conditions of the farms, the climatic conditions and the cohabitation with stray cats.

## Conclusion

The present study showed an average seroprevalence of 30.7%. The positivity found in all farms leads to the conclusion that toxoplasmosis is widespread in the majority of chicken farms, even in caged chickens. A significant association was found between *T.gondii* seroprevalence and farm type. Variations in infection rates appear to be related to differences in management practices. Intensively reared chickens are as likely to be exposed to *T. gondii* oocysts in pasture and water as free-range chickens, which benefit from more controlled management with high-quality feed and water. A higher seroprevalence was observed in yearling free-range chickens, suggesting that they pose a higher risk to the consumer. Chicken contamination is an indirect reflection of environmental contamination. For this reason, we can confirm that the prevalence of *T. gondii* in chicken is an excellent indicator for estimating the contamination of the premises where they are reared and for limiting the circulation of toxoplasma strains. Poultry products can be a source of food contamination. In addition, compliance with hygiene rules at all stages of poultry meat production (rearing, slaughter and marketing) is essential to preserve the sanitary qualities of the product and to protect the health of consumers of undercooked or uncooked meat.

## Funding

No funding was provided for this work.

## Authors’ contributions

Laila Hoummadi: Sampling, methodology, article writing and validation.

Salma Berrouch and Oussama Dehhani: Sampling and validation.

Denis Limonne and Pierre Flori: conceptualization, validation, and resources.

Redouane Moutaj and Jamal Eddine Hafid: supervision and correction of the article.

## Conflict of interest

The authors declare no conflict of interests.
